# Smallpox: The Death of a Disease and  House on Fire: The Fight to Eradicate Smallpox

**DOI:** 10.3201/eid1711.111229

**Published:** 2011-11

**Authors:** Parker A. Small

**Affiliations:** University of Florida, Gainesville, Florida, USA

**Keywords:** smallpox, disease, viruses, immunization, vaccination, eradication, Nigeria, India, USSR, Smallpox: the death of a disease, House on fire: the fight to eradicate smallpox

Smallpox eradication is one of humankind’s greatest accomplishments, but like all great successes, it was the result of many individual efforts. For >3,500, years smallpox (variola major strain of the virus) had killed, scared, or blinded most inhabitants of the world. In the past century, it killed more than half a billion persons, and death was dreadful. In large cities, it was a childhood disease infecting almost every child and killing 30% of them. In isolated smaller communities, the disease would strike periodically, killing one third of those who had not previously had smallpox. When introduced into the Western Hemisphere, it killed >80% of the native population. Smallpox changed history, impacted monarchies, and affected outcomes of wars. Its detailed history is told in Smallpox and Its Eradication by Fenner et al., and is available on the World Health Organization (WHO) website (*1*), but the story is now available in more readable forms.

Smallpox: The Death of a Disease, by D.A. Henderson, is the story from the top down by the program director, and House on Fire: The Fight to Eradicate Smallpox by William F. Foege, at that time a medical missionary in Africa, is told from the bottom up. Henderson had recruited Foege because of his prior work at the Centers for Disease Control and Prevention and public health training.

Both books are full of interesting anecdotes. Henderson’s stories largely focus on getting around WHO bureaucracy, often by bypassing it functionally, while appearing to follow it carefully. For example, vaccine was supplied immediately after an emergency request was made, but the request was also processed through WHO channels. Two years later, the emergency request was appropriately approved. Dr Foege's anecdotes focus on the challenges in the field, stopping smallpox in eastern Nigeria, and later in India. For example, when relations between eastern and western Nigeria became difficult, and persons in the capital (Lagos) would not send vaccine to continue uninterrupted immunizations, Foege stole vaccine from their warehouse. These 2 books describe dedicated, creative, and capable persons working in many countries and overcoming unpredictable obstacles.

The original plan was for mass vaccination of >80% of the population in any country that had smallpox cases. When Foege was beginning his work in Nigeria, he heard from missionaries communicating by short wave radio that there were outbreaks of smallpox in several villages. He did not have enough vaccine for mass vaccination of the region. Instead, he went to each village whose inhabitants had smallpox infections and vaccinated the inhabitants of only that village and perhaps 1 or 2 nearby villages that had frequent contact. This procedure was called surveillance and containment. As he pursued this approach, smallpox seemed to be cleared from eastern Nigeria. The civil war between the eastern and western regions caused him to evacuate for >1 year. When he returned, eastern Nigeria was still free of smallpox. This amazing result was shared with programs from other nations but met with mixed acceptance. Mass vaccination had been the accepted way.

Foege’s final proof of the effectiveness of surveillance and containment came when he was assigned as advisor to the most challenging state in India. The surveillance system there was far more complex than relying on shortwave radio reports of missionaries from isolated communities. In addition, the density and mobility of the population meant that containment had to be more rigorous. As improved surveillance detected more cases, the Indian Minister of Health wanted to return to mass vaccination, but a young, brave health officer pointed out that when a house is on fire, you pour water on that house, not in the neighborhood (thus the book title). That simple argument saved the program. On the basis of the increasing number of reported cases, the Communist Party in India accused the United States of spreading the disease. Foege had to convince Ambassador Daniel Patrick Moynihan to allow the program to continue. The last case of smallpox in India occurred in a homeless beggar on May 18, 1975. She had spent 4 days on the platform of a railroad station potentially infecting thousands. While she was there, 4,500 tickets had been sold to persons traveling to stations throughout India, prompting an intensive widespread search. Fortunately, no additional cases were detected.

Henderson tells the story of the mixed role of the USSR. Few persons realize the key role the USSR played in eradicating smallpox. The USSR was the original force behind the proposal to WHO to eradicate smallpox and provided free vaccine to much of the world. It is unlikely that eradication would have been achieved without the contributions of the USSR. Conversely, the last chapter in Henderson’s book, entitled Smallpox as a Biological Weapon, documents the inhumane posteradication activities of the USSR to produce 50–100 tons of smallpox virus per year for use as a biological weapon. The story of how this information came to light, and the subsequent response of our government to protect our nation, needs to be more widely known.

These 2 books tell the story of humankind’s most remarkable achievement, the eradication of the most dreaded infectious disease in history. I have never understood how the Nobel Prize Committee has avoided recognizing this achievement. Two men played the 2 most critical roles. Foege showed that surveillance and containment worked. Henderson helped create and then direct the program and overcame seemingly insurmountable obstacles. Perhaps the greatest obstacle was the “knowledge” that eradication was impossible, but as Foege said, “Something must be believed to be seen.” Although hundreds of thousands of persons were involved in the eradication of smallpox, Henderson provided the leadership to get it completed and Foege showed how it should be conducted, even in the most challenging of settings. It is time for their contributions to be recognized!

## References

[1] Fenner F. Henderson DA, Arita I, Ježek Z, Ladnyi ID. Smallpox and its eradication. Geneva: World Health Organization; 1988 [cited 2011 Aug 18]. http://whqlibdoc.who.int/smallpox/9241561106.pdf

